# 

**Published:** 1908-09

**Authors:** 


### BOOKS RECEIVED.

The Principles and Practice of Gynecology. By E. C. Dudley, Professor of Gynecology in the Northwestern University Medical School, Chicago. Fifth edition. Octavo, pp. 806. With 431 illustrations and 20 full page plates. Philadelphia and New York: Lea & Febiger. (Cloth. $5.00; leather, $6.00; half morocco, $6.50, net prices).

Pulsating Exophthalmos. By George E. de Schweinitz, M.D., Professor of Ophthamology in the University of Pennsylvania, and Thomas B. Holloway, M.D., Instructor in Ophthalmology. Small octavo. p. 124. Philadephia and London: W. B. Saunders Co. (Cloth, $2.00).

Pain. Its Causation and Diagnostic Significance in Internal Diseases. By Dr. Rudolph Schmidt, Assistant in the Clinic of Hofrat von Neusser, Vienna. Translated by Karl M. Vogel and Hans Kinsser, College of Physicians and Surgeons, New York. 12mo, pp. 326. Philadelphia and London: J. B. Lippincott Co. (Cloth. $3.00).

Cataract Extraction. A Series of Papers with discussions read before the Ophthalmological Section of the New York Academy of Medicine, 1907-1908. Edited by J. Herbert Claiborne, M.D., Instructor in Ophthalmology, Cornell University Medical College. Small octavo, pp. 169. New York: William Wood & Co. (Cloth, $2.00.)

Pathological Technic. By Frank Burr Mallory, M.D.. and James Homer Wright, M.D., Assistant Professors of Pathology, Harvard University Medical School. Fourth edition. Octavo, pp. 480. Illustrated. Philadelphia and London: W. B. Saunders Co. (Cloth, $3.00.)

Diseases of Infancy and Childhood. By Louis Fischer, M.D., Attending Physician to the Willard Parker and Riverside Hospitals. New York. Second edition. Octavo, pp. 979. With 403 illustrations. several in colors, and 27 full page half-tone and color plates. Philadelphia: F. A. Davis Co.

The Ready-Reference Handbook of Diseases of the Skin. By George Thomas Jackson, M.D., Chief of Clinic and Instructor in Dermatology, College of Physicians and Surgeons, New York. Sixth edition. 12mo, 737 pages, with 99 engravings and 4 plates, in colors, and monochrome. Lea & Febiger, Publishers, Philadelphia and New York, 1908. Cloth. $3.00, net.

The Principles of Pathology. Volume I., General Pathology. By J. George Adami, M.A., M.D., LL.D., F.R.S., Professor of Pathology in McGill University, Montreal. Octavo, 948 pages with 322 engravings and 16 plates Lea & Febiger, Publishers. Philadelphia and New York, 1908. (Cloth, $6.00 net.)

Consumption: How to Prevent it and How to Live With It. By N. S. Davis, A.M., M.D., Professor of Principles and Practice of Medicine, Northwestern University Medical School, Chicago. Second edition. 12mo. 172 pages. F. A. Davis Company, Publishers, Philadelphia. (Cloth, $1.00.)

Pulmonary Tuberculosis and Its Complications. By Sherman G. Bonney, M.D.. Professor of Medicine, Denver and Gross College of Medicine, Denver. Octavo of 778 pages, with 189 original illustrations, including 20 in colors and 60 *x*-ray photographs. Philadelphia and London: W. B. Saunders Company, 1908. (Cloth, $7.00; half morocco, $8.50 net prices.)

Reference and Dose Book. By C. Henri Leonard, A.M., M.D., Emeritus Professor of Gynecology in the Detroit College of Medicine. New edition. 16mo, 145 pages. The Illustrated Medical Journal Company, Detroit. (Price. 75 cents.)

